# Case Report: Untreatable Headache in a Child With Ventriculoperitoneal Shunt Managed by Use of New Non-invasive Intracranial Pressure Waveform

**DOI:** 10.3389/fnins.2021.601945

**Published:** 2021-02-10

**Authors:** Geraldo Paraguassu, Mark Khilnani, Nicollas Nunes Rabelo, Luiza D'Ottaviano Cobos, Gustavo Frigieri

**Affiliations:** ^1^Hospital Estadual Alberto Torres, Rio de Janeiro, Brazil; ^2^Bio Technology Consulting Services, Atlanta, GA, United States; ^3^Atenas Medical School, Passos, Brazil; ^4^Department of Medicine, Universidade José Do Rosário Vellano, Alfenas, Brazil; ^5^Brain4care, São Paulo, Brazil

**Keywords:** altered intracranial pressure, craniopharyngioma, hydrocephalus, non-invasive intracranial pressure monitor, ventriculoperitoneal shunt, untreatable headache

## Abstract

brain4care, a new Food and Drug Administration (FDA)-cleared non-invasive sensor that monitors intracranial pressure waveforms, was used in a 13-year-old girl who presented with untreatable headaches. The patient had a history of craniopharyngioma resection and a ventriculoperitoneal shunt placement 7 years prior to the use of the device. Secondary obstructive hydrocephalus was also a present factor in the case. The hypothesis was that due to the hydrocephalus, the child presented chronic headaches and needed constant readjustment into the ventriculoperitoneal shunt to regulate the cerebrospinal fluid inside her ventricles in order to control the patient's intracranial pressure (ICP). The device was chosen considering the risks to submit a patient into the regular invasive method to measure ICP. It was identified that the device could also indicate altered intracranial compliance due to the ratio between the P1 and P2 amplitudes (P2/P1 ratio > 1).

## Introduction

Craniopharyngiomas are classified as non-glial, benign, slow-growing tumors in the sellar/suprasellar region; there are two histologic subtypes known as adamantinomatous and papillary craniopharyngioma. The general clinical presentation consists of constant headaches and nausea and vomiting (non-specific symptoms of raised intracranial pressure—ICP); visual symptoms could be present, commonly in adults, and endocrine disturbances are normally present in the cases permanently, needing constant care (Garnett et al., 2007; Torres et al., 2020).

Secondary obstructive hydrocephalus is a common cause of the high ICP in the cases, due to the compression of the 3rd ventricle and obstruction of the interventricular foramina (foramina of Monro) or the cerebral aqueduct (Sylvian aqueduct) resulting from the growth of the craniopharyngioma. The diagnosis is based on the patient's clinical and scanning images, confirmed later through histological analysis (Garnett et al., 2007; Torres et al., 2020).

The diagnosis of hydrocephalus and shunt dysfunction, especially in children, tends to be difficult due to the common symptoms, leading to several exams, tests, and intracranial pressure measurement. ICP is commonly monitored athwart the placement of a catheter and pressure transducer into the meninges' division spaces, in other words, an invasive procedure, which brings along a risk of infections and bleedings (Ballestero et al., 2017; Bezerra et al., 2018; Frigieri et al., 2018a).

The new non-invasive ICP monitor method from brain4care is based on mechanical extensometer that can be attached through a band into the external part of the cranial bones, normally in the parietal region laterally to the sagittal suture, but it can be used in other bones such as the occipital, reporting the brain waveforms without the risks of undergoing an invasive procedure (Ballestero et al., 2017; Bezerra et al., 2018; Frigieri et al., 2018a).

## Case Report

The patient, a 13-year-old girl, presented in November 2018 to a neurosurgical clinic with severe bilateral frontal headaches in a stabbing pattern, which have started earlier that year. The child was not presenting fever. She has a medical history of craniopharyngioma and a ventriculoperitoneal adjustable shunt after a computerized axial tomography scan (CAT) revealed a suprasellar mass involving the 3rd ventricle with secondary obstructive hydrocephalus, treated 7 years prior to the consult. The patient was referred to the neurosurgical clinic after several emergency department visits, CAT scan imaging, and laboratory tests were not able to elucidate the etiology of the child's headache.

The patient's adjustable shunt regulator valve, which was a standard regulation device, was investigated, and it was noticed that it was set at the lowest setting (0.5)—the device has a range of five settings (0.5, 1.0, 1.5, 2.0, and 2.5). Each increase in the setting number increases the opening pressure that is needed to allow cerebrospinal fluid (CSF) to drain from the ventricles into the catheter, through the adjustable one-way valve, and into the peritoneal cavity.

The hypothesis was that the patient's shunt was allowing too much CSF to drain out of the brain, so the valve setting was changed from 0.5 to 1.5, allowing less CSF to drain out of the ventricles, changing the opening pressure from 1.5 to 7.0 cmH2O. After the adjustment, the patient remained asymptomatic for almost a year; however, during 2019, her symptoms returned.

**Figure 1:**
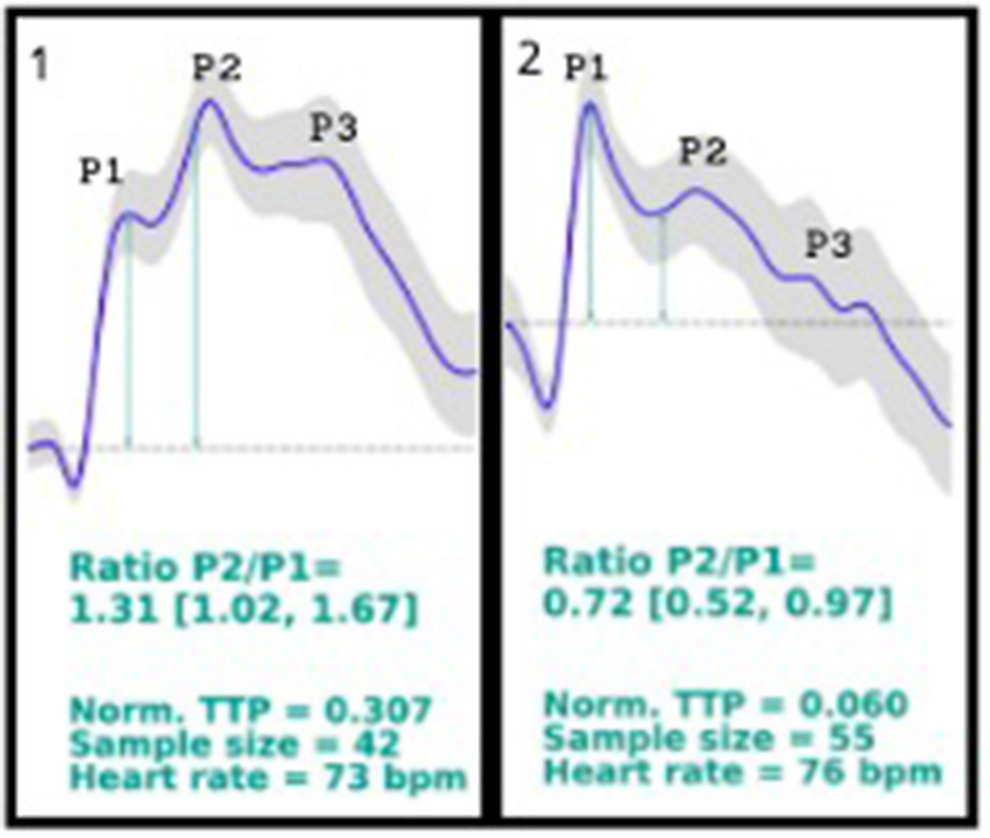
1st curve description: P2/P1 ratio = 1.31 (1.02, 1.67); norm. TTP = 0.307; sample size = 42; heart rate = 73 bpm. Patient's brain waveforms at the first time using brain4care sensor, showing a P2 peak higher than the P1 peak. 2nd curve description: P2/P1 ratio = 0.72 (0.52, 0.97); norm. TTP = 0.060; sample size = 55; heart rate = 76 bpm. Patient's brain4care sensor waveform record after the shunt valve was changed from 2.5 to a 1.0 setting. The P1 peak is higher than the P2 peak.

On October 2019, the company responsible for brain4care (B4C), a non-invasive Food and Drug Administration (FDA)-cleared device, which monitors intracranial waveforms through sensors and platforms in patients with suspected elevated intracranial pressure, was contacted. It was noticed that the patients monitored by the non-invasive B4C displayed similar morphology waveforms as standard invasive intracranial pressure sensors, resulting in characteristic peaks. On October 14th, the B4C was placed in the patient's head to record the brain waveforms; however, the patient's results showed a higher P2 peak than the usual higher P1 waveform peak (Figures 1, 1st curve).

As the B4C sensor data displays waveform shape and not the absolute intracranial pressure value, it was suspected that the patient's brain had poor compliance due to high intracranial pressure, considering that the location of the patient's P1 and P2 peaks appeared to be reversed. In parallel studies, it is being seen that patients with altered intracranial compliance presents a P2/P1 ratio > 1.

The shunt valve was checked to decipher the outflow pressure setting; considering the patient's previous improvement, the adjustable valve had been setted from 1.5 to the highest (2.5), which resulted in a cranial hypotension, thus not allowing enough CSF to drain out of the ventricles. The valve setting was lowered to 1.0, which requires 3.5 cmH2O opening pressure, allowing more CSF to drain out, and the patient became asymptomatic after the change.

The B4C sensor was placed again in the patient's scalp, in the same position, registering new waveforms (Figures 1, 2nd curve), showing the P2 peak lower than the P1 peak.

## Discussion

The use of the new device to measure the intracranial pressure through a non-invasive method avoiding the patients' exposure into several possible complications is a groundbreaking technique. The case is remarkable given that the patient was a 13-year-old girl with a history of undergoing brain surgery, which was already a risk, and a severe, untreatable headache that constantly caused discomfort to the patient. Considering that every exam and test led to no answer, the hypothesis of unregulated intracranial pressure (ICP) as a result of shunt malfunction and hydrocephalus along with poor brain compliance could previously only be confirmed through an invasive procedure, putting the patient under risks, in order to relieve the pain.

Monitoring the ICP is essential in neurology and neurosurgery, as it could indicate several diagnoses and prognoses in different neuropathologies. The ICP is the result of the dynamics between three components, the brain's structures, the cerebrospinal fluid (CSF), and the vascular component (circulating blood in the brain), being responsible to maintain the ICP normal values. The graphic result of the ICP waveform measurement consists in three characteristic peaks: P1, representing the arterial pressure being exerted from the choroid plexus to the ventricle, known as the *percussion wave*; P2, associated to the brain compliance, known as the *tidal wave*; and P3, which is the *dicrotic wave*. Under normal circumstances, the waves are presented as P1 > P2 > P3 (Cabella et al., 2016; Ballestero et al., 2017; Frigieri et al., 2018b).

The device uses sensors coupled to the brain that captures the intracranial waves and pulses based on the force exerted in the skull caused by the expansions and retractions in the volume, converting the changes into numbers and resulting in graphic results. Non-invasive techniques usually face complications based on anatomical diversities; however, the B4C device has been tested and compared to invasive techniques, and it has been proved that it can indicate altered intracranial compliance when the P2/P1 ratio is analyzed. In the case reported, a P2/P1 ratio > 1 was encountered, which indicates poor intracranial compliance, and by the analysis of the case, it can be concluded that it was secondary to the hydrocephalus and shunt malfunction, leading to the regulation of the shunt and relief of the pain (Cardim et al., 2016; Vilela et al., 2016; Ballestero et al., 2017; Bollela et al., 2017).

## Conclusion

The new brain4care monitor is considered to be an important new tool to investigate high intracranial pressure suspected cases without using current invasive methods.

Through the analysis of the waveforms and known patterns (P1 > P2 > P3), the graphic result, accompanied by the patient's history and clinical data, indicates altered brain compliance. This can show a way to deal with the case and find a path to improve quality of life without exposing the patient to possible hemorrhages and infections. Numerical waveform analysis is another positive point of the method, since it allows the medical team a more objective and clear analysis, making the process observer-independent.

## Data Availability Statement

The original contributions presented in the study are included in the article/supplementary material, further inquiries can be directed to the corresponding author/s.

## Ethics Statement

Ethical review and approval was not required for the study on human participants in accordance with the local legislation and institutional requirements. Written informed consent to participate in this study was provided by the patient/participants legal guardian/next of kin. Written informed consent was obtained from the minor(s)' legal guardian/next of kin for the publication of any potentially identifiable images or data included in this article.

## Author Contributions

All authors listed have made a substantial, direct and intellectual contribution to the work, and approved it for publication.

## Conflict of Interest

GF was employed by the company Brain4care and MK was employed by the company Bio Technology Consulting Services. The remaining authors declare that the research was conducted in the absence of any commercial or financial relationships that could be construed as a potential conflict of interest.

## Abbreviations

FDA: Food and Drug Administration
ICP: intracranial pressure
CSF: cerebrospinal fluid
CAT scan: computerized axial tomography scan
B4C: brain4care.
